# Three perspectives on the molecular basis of hypercontractility caused by hypertrophic cardiomyopathy mutations

**DOI:** 10.1007/s00424-019-02259-2

**Published:** 2019-02-15

**Authors:** James A. Spudich

**Affiliations:** 1 Department of Biochemistry, Stanford University School of Medicine, Stanford, CA 94305 USA; 2 Cardiovascular Institute, Stanford University School of Medicine, Stanford, CA 94305 USA

**Keywords:** β-Cardiac myosin, Hypertrophic cardiomyopathy, Interacting heads motif, Super-relaxed state, Load

## Abstract

Several lines of evidence suggest that the primary effect of hypertrophic cardiomyopathy mutations in human β-cardiac myosin is hypercontractility of the heart, which leads to subsequent hypertrophy, fibrosis, and myofilament disarray. Here, I describe three perspectives on the molecular basis of this hypercontractility. The first is that hypercontractility results from changes in the fundamental parameters of the actin-activated β-cardiac myosin chemo-mechanical ATPase cycle. The second considers that hypercontractility results from an increase in the number of functionally accessible heads in the sarcomere for interaction with actin. The final and third perspective is that load dependence of contractility is affected by cardiomyopathy mutations and small-molecule effectors in a manner that changes the power output of cardiac contraction. Experimental approaches associated with each perspective are described along with concepts of therapeutic approaches that could prove valuable in treating hypertrophic cardiomyopathy.

## Introduction

The most common form of inherited heart disease is hypertrophic cardiomyopathy (HCM), which affects more than 1 in 500 individuals [32, 54, 74]. It is characterized by left ventricular hypertrophy in the absence of predisposing conditions, and ultimately results in decreased left ventricular chamber volume. Genetically based HCM has been shown to involve the sarcomeric proteins directly [73]. Out of hundreds of known HCM mutations, ~ 35% occur in human β-cardiac myosin, ~ 35% in cardiac myosin-binding protein-C (MyBP-C; consisting of 13 domains, C0, PA, C1, M, C2, C3, C4, C5, C6, C7, C8, C9, and C10 [30]), and most of the rest are distributed amongst other sarcomeric proteins, especially in the troponins and tropomyosin [47, 102]. There are reported to be more than 300 pathogenic HCM mutations in β-cardiac myosin [13, 18, 72, 94], and most of these are missense mutations in the globular head domain [18]. Interestingly, most of the mutations in MyBP-C result in truncations of the protein and lead to haploinsufficiency of the protein [30, 55, 92], while HCM mutations in myosin are missense mutations that alter the functionality of the protein. Since human β-cardiac myosin and MyBP-C are the primary carriers of HCM mutations, there is likely to be a mechanistic link between them, which I discuss further below.

The earliest manifestations of genetically based HCM are impaired diastolic dysfunction with either preserved systolic function or even hypercontractility as judged by echocardiography [33, 35, 36]. These functional characteristics are evident before any hypertrophy is observed. To be sure, HCM is a heterogeneous condition with differences in specific mutation, penetrance, allelic imbalance, cardiac geometry, and genetic and environmental modifiers, all contributing to the final outcomes for any individual patient, and it is therefore difficult to correlate disease phenotype solely with in vitro interactions of myosin with actin. Nonetheless, unlike dilated cardiomyopathy which results in a hypocontractile heart, increases in left ventricular ejection fraction are seen in genotype-positive HCM patients compared to controls in early-stage patients [14, 29, 36], and my laboratory is working on the hypothesis that HCM mutation–induced hypercontractility leads to activation of signaling pathways that cause hypertrophy of the heart and later fibrosis and myofilament disarray (but see the “Conclusions” for other considerations). Supporting this view are the findings that chronic treatment with isoproterenol and omecamtiv mecarbil, both activators of heart contractility, leads to ventricular hypertrophy [22, 53]. Also supporting this view are studies using inhibitors of cardiac contractility. If the earliest manifestation of HCM were hypocontractility of the heart, the disease would be exacerbated by further inhibition by small-molecule inhibitors. But just the opposite is seen. Early treatment of HCM-mouse models with the recently developed myosin inhibitor mavacamten prevents development of left ventricular hypertrophy and fibrosis, and even reverses existing hypertrophy in older HCM-mutant mice [22, 27]. Mavacamten has been also used in human clinical trials with significant reduction in left ventricular outflow gradient in patients with obstructive HCM [40]. These findings support the concept that a hypercontractile state of the sarcomere caused by HCM mutations is the unifying initial parameter that leads to pathologic left ventricular hypertrophy and fibrosis.

Hence, a pivotal question is, what is the molecular basis of the hypercontractility seen in HCM patients? If the mechanism(s) generating the hypercontractility were understood, one could look for small-molecule effectors that bind directly to the mutated sarcomeric protein of interest, myosin in the case of HCM-myosin mutations, and reverse the effects of the HCM mutations, potentially by the same mechanisms that cause the hypercontractility in the first place. Cardiac contraction is complex, involving highly specific kinetics of each step in the chemo-mechanical ATPase cycle, load-dependent changes in behaviors of contraction, and multiple layers of regulatory mechanisms to assure optimal performance of the heart. This means that there are multiple perspectives for thinking about how hypercontractility is achieved mechanistically. This review focuses primarily on β-cardiac myosin HCM mutations, the most common type. Here, I describe three perspectives on how to think about hypercontractility of the sarcomere from a molecular point of view.

## The first perspective: hypercontractility results from changes in the fundamental parameters of the actin-activated β-cardiac myosin chemo-mechanical ATPase cycle

### Hypercontractility results from an increase in the population of myosin heads strongly bound to actin in a force-producing state

**Fig. 1:**
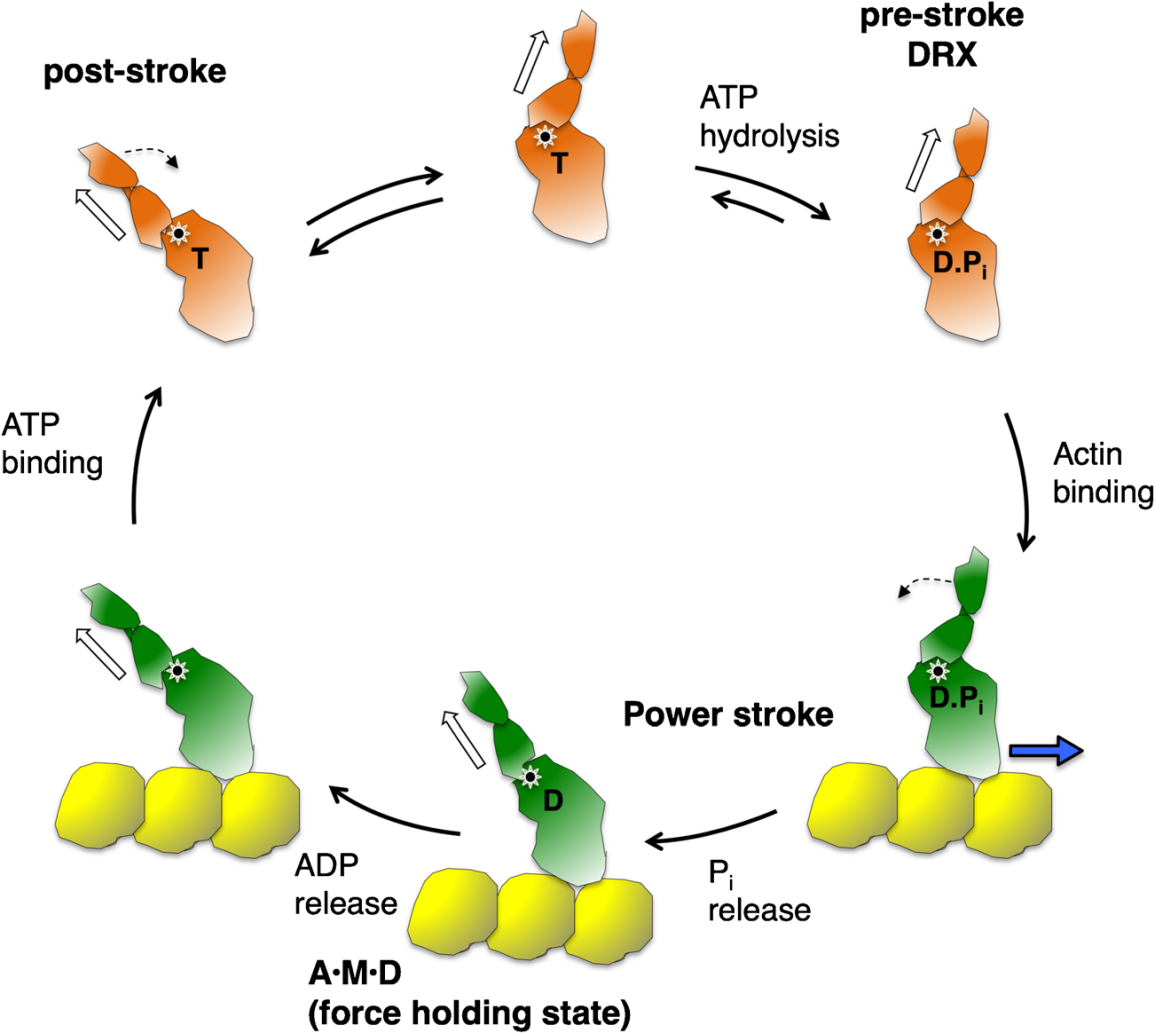
Chemo-mechanical cycle of the interaction of myosin heads with actin. The pre-stroke S1 with bound ADP (D) and inorganic phosphate (Pi) binds to actin (yellow); while bound to actin, the lever arm swings to the left about a fulcrum point (black dot on white star), moving the actin filament to the right (bold blue arrow) with respect to the myosin thick filament; ADP release allows a further small stroke to the post-stroke position and frees the active site for binding of ATP (T); ATP binding weakens the interaction of the S1 to actin; ATP hydrolysis locks the head into the pre-stroke state, which when functionally accessible for interaction with actin is in a disordered relaxed (DRX) state

Figure 1 shows the fundamental kinetic steps in the actin-activated myosin chemo-mechanical ATPase cycle, where the ADP·Pi-bound pre-stroke state represents the conformation of the myosin head in a disordered relaxed (DRX) state waiting to bind to actin upon activation of contraction. The ATPase turnover rate of this state for β-cardiac myosin is ~ 0.03 s^−1^. Actin activates this rate by enhancing Pi and ADP release by two orders of magnitude (~ 3 s^−1^). The A·M·D state represents the ADP-bound myosin head in a force-producing state bound to actin. One point of view of the first perspective is that changes in one or more of these kinetic steps due to HCM mutations lead to more myosin heads being in the A·M·D state or remaining in the A·M·D state longer than normal, resulting in an increase in ensemble force in the muscle. If one measures all the kinetic steps of the actin-activated myosin ATPase cycle, one can model the percentage of heads in the A·M·D state during systolic contraction, a gain of the A·M·D population due to HCM mutations signifying hypercontractility. These measurements involve stopped flow experiments to obtain kinetic parameters in the millisecond time range. An example of this approach can be found in recent work [90], where a clear distinction could be made between four dilated cardiomyopathy (DCM, which results in a dilated heart) mutations (I201T, R237W, S532P, and F764L), which cause hypocontractility of the heart and resulted in a decrease in A·M·D population, and an HCM mutation (R453C), which resulted in an increase in A·M·D population. In favor of this approach is that a thorough analysis of the kinetics of the cycle is performed. The difficulty with this approach is that many distinct measurements are needed, many of those measurements involve perturbations of the system (e.g., the use of fluorescent analogs like mantATP, which do not necessarily accurately mimic ATP), and not all steps can be directly measured and need to be estimated. Furthermore, one does not expect increases in A·M·D population of more than 10–30% since the primary contractility changes in HCM are relatively small, and the individual errors in the kinetic measurements made can add up, making it difficult to be certain of a small overall change in contractility. Finally, as discussed below, this point of view does not take into account possible changes in the intrinsic force (*F*_int_) of the motor or that muscle mechanics is load dependent.

### Hypercontractility results from an increase in at least one of the three fundamental parameters, ATPase activity, velocity, and intrinsic force

**Fig. 2:**
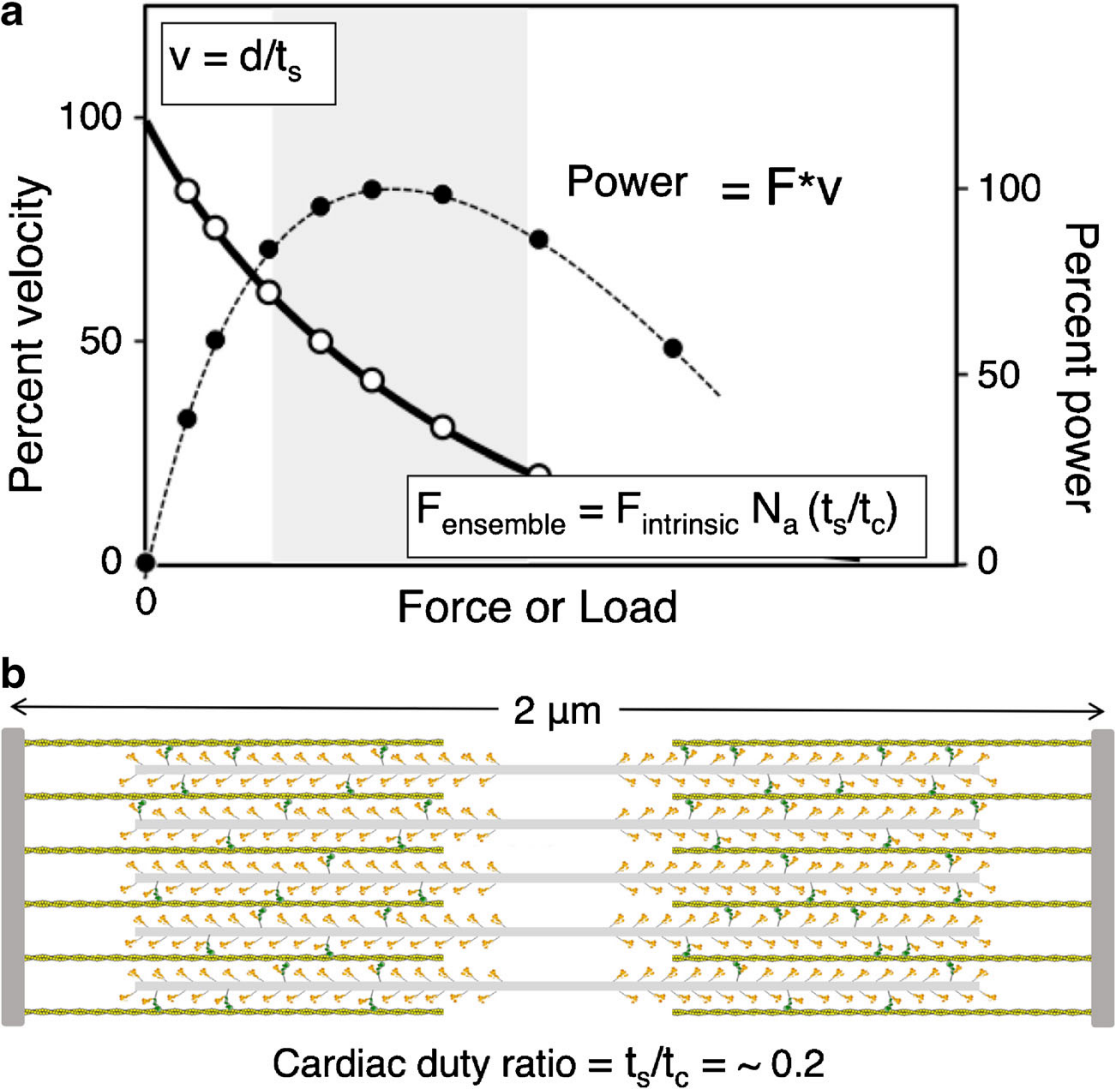
The force-velocity curve for muscle contraction and schematic of the cardiac sarcomere in its relaxed state. **a** The force-velocity curve (hyperbolic solid line, open circles) refers to how the velocity (v) decreases as a function of the load (F) imposed on the contractile machinery. The shape of the curve depends on how much force the contractile machinery can produce. At any velocity along the curve, the ensemble force of the system matches the equal and opposite load imposed. Power output is the force times the velocity at every point along the curve (dashed line, solid circles). The gray zone is the region of highest power output. **b** Schematic description of a cardiac sarcomere drawn to scale, depicted at its resting length just beginning its contraction. The sarcomere is 2 μm long, the myosin bipolar thick filaments are 1.6 μm long, and the actin filaments are 0.8 μm long. A duty ratio (the fraction of the functionally accessible heads in a strongly bound force-producing state (green)) of ~ 0.2 is depicted

A second point of view of the first perspective derives from the force-velocity relationship that is fundamental to the power output of the heart (Fig. 2a). At very low load, contraction velocity (v) is high, and velocity decreases as load on the heart (blood pressure) increases. At any load (or force that myosin produces to overcome that load), the force times the velocity is the power output. The ensemble force that myosin produces in the sarcomere is *F*_ens_ = *F*_int_·*N*_a_·*t*_s_/*t*_c_, where *F*_int_ is the intrinsic force of the myosin molecule, *N*_a_ is the number of functionally accessible heads for interaction with actin, and *t*_s_/*t*_c_ is the duty ratio that is determined by the kinetic parameters of the ATPase cycle, where *t*_s_ is myosin’s strongly bound state time to actin and *t*_c_ is the total cycle time of the actin-activated myosin ATPase cycle. The cardiac duty ratio, or the fraction of functionally accessible myosin heads in their strongly bound state to actin at any moment during systole, on average, is ~ 0.2 (Fig. 2b). According to this point of view, hypercontractility due to HCM mutations is likely to be seen as an increase in power, and the parameters most likely increased by the mutation are the velocity, the intrinsic force of myosin, and the duty ratio. Changes in *t*_s_ should affect the velocity (v) of contraction since v is related to *d*/*t*_s_, where *d* is the myosin stroke size. Changes in *t*_c_ mean the *k*_cat_ of the ATPase cycle has changed, where *t*_c_ = 1/*k*_cat_. Thus, HCM-mutant myosin could be associated with increases in ATPase activity, velocity (measured with an in vitro motility assay), and/or *F*_int_ of myosin (measured with a laser trap). In favor of this approach is that the fundamental parameter *F*_int_ is taken into consideration, and all three of these parameters are accurately measurable and capture the essence of how muscle performs. The primary negative of this approach is that the measurements are not made under varying loads, and we know that muscle mechanics is load dependent (see perspective 3 below). Nonetheless, one would expect that the measurements at near zero load would still reflect changes due to HCM mutations.

Consistent with this point of view, all three parameters (ATPase activity, velocity, and *F*_int_) increase significantly when human β-cardiac myosins carrying the early-onset HCM mutations D239N and H251N are compared to wild-type (WT) myosin [2]. These results are consistent with earlier studies using mouse α-cardiac myosin, which showed that R403Q and R453C myosins showed substantial increases in either ATPase activity, velocity, or intrinsic force compared to WT mouse α-cardiac myosin [24, 58, 89]. From these early studies, the primary hypothesis for the molecular basis of increase in power output due to HCM mutations had been that such increases explain the hypercontractility seen clinically. However, when human β-cardiac myosins containing adult-onset HCM mutations were studied, the picture was not so clear. For each of R403Q-, R453C-, R719W-, and R723G-human β-cardiac myosins, there are both small increases and small decreases in these three parameters such that it is difficult to determine whether the myosin is rendered hypercontractile or hypocontractile [45, 59, 78]. And for R663H- and G741R-myosins, there were no observable changes in any of these three parameters compared to WT human β-cardiac myosin [45, 71]. These findings led to perspective 2.

## The second perspective: hypercontractility results from an increase in the number of functionally accessible heads in the sarcomere for interaction with actin

### The myosin mesa plays a critical role in regulating the number of myosin molecules functionally accessible for interaction with actin

**Fig. 3:**
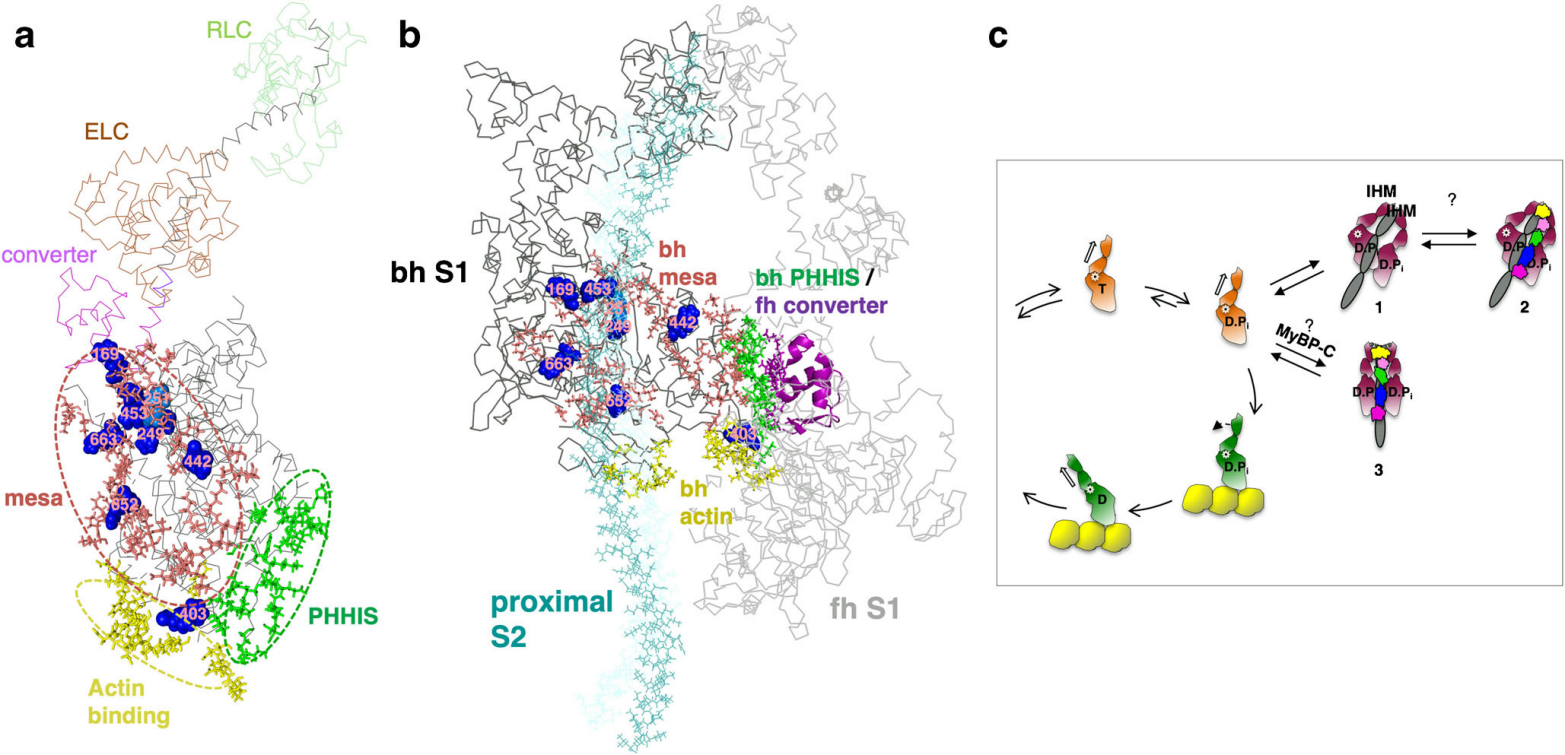
Human β-cardiac myosin structural models. **a** PyMol homology model of human β-cardiac myosin S1 in the pre-stroke state (HBCprestrokeS1; downloadable at http://spudlab.stanford.edu/homology-models/). Domains of the heavy chain are the mesa surface (salmon), the actin-binding surface (yellow), the primary head-head interaction site of the blocked head in the IHM (green), and the converter (purple). The ELC (brown) and the RLC (green), bound to the light-chain binding domain (gray) of the heavy chain, constitute the lever arm. The positions of the blocked head mesa Arg residues 169, 249, 251, 403, 652, and 663, which when mutated cause HCM, are shown in blue. Modeling was done as described previously [60, 88]. **b** Homology model of the IHM off state of human β-cardiac myosin (MS03; downloadable at http://spudlab.stanford.edu/homology-models/). The motor domains are dark gray (blocked head; bh) and light gray (free head; fh), the ELCs and RLCs are in shades of gray, and proximal S2 is in cyan. Otherwise, the color scheme is as in (**a**). **c** Two possible sequestered states of MyBP-C-bound HMM are shown. One possible state further stabilizes the normal folded-back sequestered state (depicted here as the IHM configuration) while the other is a distinctly different folded sequestered state. Both MyBP-C-bound myosin structural states are highly theoretical and serve as working hypotheses going forward

On December 14, 2014, I went to bed puzzling over our observations that the common paradigm of increased levels of the fundamental parameters ATPase activity, velocity, and *F*_int_ was not holding up to explain hypercontractility derived from HCM mutations in myosin. My wife Anna encouraged me to “just for this one night stop thinking about myosin and read this murder mystery based in the Southwest called The Haunted Mesa by Louis L’Amour.” She knew I would like it because I like murder mysteries, but more importantly I love flying over Monument Valley and viewing its many flat-surfaced mesas from the perspective of a small aircraft at a few thousand feet above the ground. I agreed and read the first twenty pages and fell asleep. My wife’s intentions were good, but I woke at 5:30 out of a dream—I dreamt that myosin has a mesa! I got up and turned the tea kettle on and pulled up the myosin molecule on PyMol, because all of us myosinologists remember the original characterization of myosin globular Subfragment 1 (S1; the globular head of myosin consisting of the amino-terminal ~ 220 kDa of the myosin heavy chain plus the essential light chain (ELC) and regulatory light chain (RLC)) by Ivan Rayment and his colleagues [67] as having the shape of a whale, with a relatively flat back. Within an hour, I discovered that this flat “myosin mesa” surface was unusual in that its residues were extraordinarily highly conserved for cardiac myosins from mouse to man. The crowning realization that the mesa was of importance was that as I viewed where the dozen or so HCM mutations that my colleague, Kathleen Ruppel, had meticulously chosen for us to study because they were clearly pathogenic, they mostly fell on the mesa surface itself, with the majority of them being conversions from Arg residues (Fig. 3a). Remembering that there is another factor in the equation for ensemble force that we were ignoring, the number of myosin heads functionally accessible for interaction with actin (*N*_a_), I imagined the positively charged cluster of Arg residues acting like a docking platform for another protein that might be sequestering the myosin heads in an inactive state, setting the value of *N*_a_ below the total number of myosin heads in the cardiac sarcomere. I proposed a possible unifying hypothesis, the mesa hypothesis [79], that most HCM mutations were weakening this sequestered state, which leads to an increase in *N*_a_ and thereby causing the hypercontractility seen clinically.

It is important to distinguish between changes in *N*_a_ and changes in the duty ratio *t*_s_/*t*_c_. *N*_a_ is the number of heads that are functionally available for interaction with actin. The duty ratio is the fraction of *N*_a_ that is engaged in the strongly bound force-producing step at any moment during systolic contraction. Its value is set by the kinetic parameters of the actin-activated myosin ATPase cycle that determine *t*_s_ and *t*_c_, which are independent of *N*_a_. Thus, the actual number of heads engaged in the strongly bound force-producing state can be altered either by changing *N*_a_ or the duty ratio, or both.

I proposed that myosin-binding protein-C (MyBP-C) is a good candidate for binding to the mesa and sequestering heads [79], reducing the value of *N*_a_, for four reasons: (1) MyBP-C is closely associated with the myosin heads in the sarcomere, with its C-terminal domain binding to light meromyosin (LMM; the portion of the myosin rod which assembles to create the thick filament) [20, 81]; (2) a structural link between the myosin mesa and MyBP-C would go along with the fact that most HCM mutations lie in these two proteins; (3) the C1-C2 fragment of MyBP-C alters the structure of thick filaments in demembranated trabeculae from rat heart muscle in a manner consistent with inhibiting them [43]; (4) most importantly, there was already strong evidence for MyBP-C interaction with other domains of myosin S1 aside from LMM. Specifically, examination of mixtures of MyBP-C with HMM and Subfragment 2 (S2; the first ~ 40% of the coiled-coil rod of myosin) in the analytical ultracentrifuge showed that there is a binding site for C-protein in the S2 region of the tail [82], the C0 domain interacts with the RLC of myosin [66], and the C1-C2 fragment interacts with the proximal part of S2 [28, 31]. Furthermore, using a combined NMR and mutagenesis approach, Ababou et al. [1] placed the C1 domain of MyBP-C on top of the S1-S2 hinge. They argued that MyBP-C binding in this location could potentially influence the positioning of the S1 heads and to adjust the position of the S1 heads and thus influence muscle contraction. They postulated that the C1 may remain bound to the myosin heads even when other MyBP-C domains bound to the head let go upon MyBP-C phosphorylation, and that the MyBP-C may maintain a loose tether to the heads even as they reach across the gap to interact with actin. This would bring the MyBP-C to the actin for interactions that activate the thin filament [30]. Thin filament interactions with MyBP-C have been extensively studied, especially by Harris and colleagues [16, 30, 48, 57, 75, 91], and reviewed in other articles in this special edition. I focus here on myosin association of MyBP-C.

Other protein surfaces that are near the myosin mesa are titin, which is also associated with myosin thick filaments along their length, and the proximal part of S2 (proximal S2). Thus, in addition to MyBP-C, both titin and the proximal S2 are possible candidates for interaction with the mesa [80]. I therefore imagined a folded-back myosin structure with the mesa interacting both with proximal S2 and MyBP-C. The myosin heads would presumably be in their pre-stroke state (with ADP and Pi in the nucleotide pocket), before actin interaction had a chance to enhance the release of these products of ATP hydrolysis, and could easily fold back onto their own tail with melting of a few residues in the first part of the coiled-coil S2 domain. Myosin HCM mutations could destabilize the putative folded structure, increasing the number of functionally accessible heads for interaction with actin (*N*_a_) and thereby causing the hypercontractility seen clinically. I was not thinking specifically about the known interacting head motif (IHM) [8, 96, 100] (Fig. 3b) at the time, although as discussed below, the IHM is likely to be the sequestered state of myosin heads in the absence of MyBP-C (Fig. 3c, structure 1), and the IHM configuration might or might not turn out to be the structural basis of the putative myosin-MyBP-C structural complex.

The hypothesis that increases in *N*_a_ is the primary cause for the hypercontractility seen clinically was even more attractive since it could explain the ~ 35% of HCM mutations that are found in MyBP-C, most of which are truncation mutations that likely lead to haploinsufficiency [12, 30, 95]. Assuming that the mesa was a site of binding of MyBP-C, ~ 15% of myosin heads in the sarcomere (since the ratio of MyBP-C:myosin in the sarcomere is ~ 1:6) [10, 62] could be further sequestered into an even more inaccessible folded state (Fig. 3c, structure 2) perhaps saved for more extreme athletic needs (the IHM configuration is illustrated here, and while likely to be the structural state of interest, additional structural studies with purified β-cardiac myosin are needed to establish this for regulation of heart function, as discussed further below). Alternatively, MyBP-C could sequester myosin heads in a different structural state from that occurring in the absence of MyBP-C (Fig. 3c, structure 3). MyBP-C-sequestered heads from this putative distinct off state could be more easily accessed for contraction than those in structure 2, rather than less accessible. Either way, haploinsufficiency for MyBP-C could result in a shift in the equilibria shown in Fig. 3 in the direction of free heads, increasing *N*_a_ and accounting for hypercontractility seen clinically due to MyBP-C mutations. Thus, I saw this structural concept involving the myosin mesa as a possible unifying hypothesis for the molecular basis of hypercontractility in HCM, both for myosin and MyBP-C HCM mutations [79].

It should be emphasized that one should not expect HCM mutations to result in a massive shift in these proposed equilibria between sequestered and free heads. HCM is generally hardly noticed by patients in the first decades of life. Large perturbations in the equilibrium are probably embryonic lethal. Since the ratio MyBP-C:myosin in the sarcomere is ~ 1:6, MyBP-C could only be sequestering ~ 15% of myosin heads in an off state. A heterozygotic MyBP-C mutation that results in truncation and depletion of half of the MyBP-C would maximally add ~ 7% more heads to the pool for actin interaction. This small change may be the reason that MyBP-C HCM mutations are not generally as severe pathogenically as myosin HCM mutations [15, 61], but in general, a shift in the equilibrium in the 5–30% range may be all that occurs in the average clinical case.

In support of this unifying hypothesis, examination of hundreds of known HCM mutations confirmed that the myosin mesa is one hotspot for HCM mutations and the proximal portion of myosin S2 is another [37]. Strikingly, while the mesa surface HCM sites are largely positively charged Arg residues, the proximal S2 sites are largely negatively charged Glu or Asp residues [37]. This juxtaposition of charges could lead to an electrostatic interaction holding the heads in a sequestered state [7, 37, 60, 68, 88]. Thus, these HCM-enriched surface regions are particularly noteworthy of study. Interestingly, recent work [50] showed that incident atrial fibrillation is associated with HCM mutations in β-cardiac myosin, and that the incidence of new-onset atrial fibrillation was considerably higher in those patients with mutations in the HCM-enriched surface regions compared to those with mutations in other regions of myosin.

As already mentioned, the concept that HCM mutations could alter a sequestered state of myosin heads is not new. Early suggestions involved the asymmetric folded-back state of myosin known as the IHM (Fig. 3b), first described by Wendt et al. [96, 97] from high-resolution reconstructions of purified smooth muscle heavy meromyosin (HMM; consisting of the two S1 domains attached to S2), and later implicated in many other myosin types [5, 8, 49, 99, 100]. The first of such mappings of HCM mutations was done on proximal S2 by Blankenfeldt et al. [11], using the tarantula skeletal muscle IHM structure of Woodhead et al. [100]. They showed that the proximal S2 HCM mutations R869C and R870H probably affect the structural integrity of proximal S2, while several others lead to charge inversion. Subsequently, other investigators have mapped “HCM residues” on the IHM structure and also suggested that they may be altering the IHM structure and leading to hypertrophic cardiomyopathy [1, 58, 93, 2, 7, 37, 45, 60, 68].

A note of caution here is one must be careful in describing which putative HCM mutations are clearly causative of HCM because in many cases HCM mutations are reported from a single individual or from an insufficient number of individuals to be certain that the “HCM mutation” is not simply a normal variant in the population. One should consult the Sarcomeric Human Cardiomyopathy Registry (SHaRe), a multi-center, international repository of clinical and laboratory data on individuals and families with genetic heart disease developed as a collaboration with several world-leading cardiovascular centers, which classifies putative HCM mutations as pathogenic, likely pathogenic, and benign (https://theshareregistry.org) [34, 37, 50].

A second cautionary note is that while purified smooth muscle HMM has been shown to form an IHM structure by high-resolution electron microscopy [96, 97], most studies related to the IHM structure are based on low-resolution (≥ 2 nm) EM reconstructions from isolated myosin thick filaments (for reviews, see [6, 88]). It is imperative that the high-resolution structure of the folded-back sequestered state of human β-cardiac myosin be determined. Only then can we be confident about relating HCM mutations to changes in the structure, as has been emphasized earlier [88]. Nonetheless, the IHM state, derived from smooth muscle HMM EM reconstitutions [96, 97] and first described as the probable sequestered state in isolated thick filaments of tarantula skeletal thick filaments [100], remains the best current model for the folded-back sequestered state of human β-cardiac myosin [5].

In support of the IHM model for human β-cardiac myosin, or something close to it, mapping of the mesa residues onto the IHM structure [60] strikingly showed the “blocked head” mesa cradling proximal S2 (Fig. 3b), while a cleft between the proximal S2 and the “free head” would allow binding of MyBP-C (Fig. 3c, structure 2). The IHM-sequestered model is also an attractive sequestered state for human β-cardiac myosin in that the third hotspot for myosin HCM mutations is the converter domain [7, 37, 68], which in the IHM structural model is a primary interaction domain on the free head for interaction with a primary head-head interaction site (PHHIS) on the blocked head (Fig. 3a, b). Nonetheless, high-resolution structures of the folded-back sequestered state with purified β-cardiac HMM are in high demand. The proposed structures of a complex between the C0-C2 fragment of MyBP-C and the IHM folded-back structure [60] are highly speculative, as already emphasized [60, 88]. Also, as already mentioned, it is possible that the MyBP-C bound heads are in a somewhat different sequestered state than the heads assume in the absence of MyBP-C (Fig. 3c, structure 3). The putative MyBP-C-myosin complex could even be a more open sequestered structure. Thus, determination of even a low-resolution structure of a MyBP-C-β-cardiac HMM complex using the two purified proteins is a high priority in the field.

Related to perspective 2 is the concept of a super-relaxed (SRX) state, first introduced by Roger Cooke and his colleagues [38]. They defined a myosin state in skinned muscle fibers that has an extremely slow ATP turnover rate (~ 0.003 s^−1^). This is 1000 times slower than the actin-activated myosin ATPase rate and 10 times slower than the normal basal ATPase rate of myosin alone. Hence, it was given the name the super-relaxed state. It is estimated that nearly 50% of the myosin heads in the sarcomere may be in this state [38]. The SRX has generally been equated to the IHM structural state, but experimental evidence for this is needed. Recently, it has been shown that a folded-back sequestered state of β-cardiac myosin has the reduced ATPase activity of the SRX [9], but I use the term “folded-back sequestered state” rather than the IHM state here and in what follows to emphasize that the detailed structure of this state for purified human β-cardiac myosin has not been determined. The IHM state is the best current model [5], and I use it as a model in most of the figures in this review. It must be emphasized, however, that the definition of SRX is a form of myosin with an extremely low ATPase turnover rate [38]. Surprisingly, even sS1, which has no proximal S2 to fold back onto, can exist in a state with the SRX level of ATPase [9, 69], which shows that SRX cannot be exclusively defined as a folded-back state.

The unifying hypothesis that the majority of myosin HCM mutations weaken a sequestered state, increasing the number of heads functionally accessible for interacting with actin, thus causing the hypercontractility seen clinically, is attractive, and experimental evidence is accumulating in favor of it. First, when mapping hundreds of myosin HCM mutations on the molecule, the hotspots that are noted are the major intramolecular interaction domains of the IHM structure [7, 37, 68]. As predicted by the IHM folded-back sequestered model, the proximal S2 domain of myosin has been shown to bind in trans to short S1 (sS1; ending at residue 808 for human β-cardiac myosin and therefore containing the ELC but not the RLC) [60], and the mesa HCM mutations R249Q, H251N, and R453C weaken this binding [2, 60]. Weakening of this binding is also true for the proximal S2 D906G HCM mutation [60]. Using functional assays for changes in *N*_a_, the mesa HCM mutations R249Q, H251N, R403Q, and R663H cause an increase in *N*_a_ [3, 71] as does the converter HCM mutation R719W and the PHHIS HCM mutation D382Y [3]. Importantly, no example of an HCM mutation that appears to decrease *N*_a_ has been reported. These results support both the unifying hypothesis for hypercontractility [79] and the IHM model as the folded-back sequestered state [5, 100]. As a control, the HCM mutation I457T, which is not at any of the interacting surfaces involved in IHM formation, does not appear to change *N*_a_. This mutation is buried in the transducer of the catalytic domain and I457T human β-cardiac myosin, in keeping with perspective 1, shows significant increases in velocity and ATPase [3], which may account for hypercontractility in patients carrying this mutation.

Assuming MyBP-C is involved in sequestering myosin heads in an off state, the work of Gruen and Gautel [28] is highly relevant. They showed by direct binding experiments with purified proteins that the proximal S2 HCM mutations R870H and E924K lead to a dramatic loss of binding of the C1-C2 fragment of MyBP-C to proximal S2. In a reciprocal study, as judged by yeast two-hybrid analysis, the MyBP-C HCM mutation E258K in the C1 domain of MyBP-C abolishes the interaction between the N-terminal C1-C2 fragment of MyBP-C and proximal S2 [23]. More recently, the myosin mesa involvement was demonstrated with the finding that the mesa HCM mutation R403Q decreases dramatically the affinity of the C0-C7 fragment of MyBP-C to 25-hep HMM (a two-headed construct ending at residue 1016 for human β-cardiac myosin), again with direct binding experiments with purified proteins [71].

Complementary to the biochemical studies with purified proteins, studies using cardiac muscle fibers from pig models showed that the R403Q HCM-myosin mutation causes a decrease in the SRX state in muscle fibers [9]. Furthermore, examination of the R663H HCM-myosin mutation in fibers from hearts of multiple HCM patients showed reduced SRX state [9], although others have not seen such a change in HCM-myosin patient samples [56].

A significant decrease in the proportion of myosin heads in the SRX state was seen in homozygous cMyBP-C (cardiac MyBP-C) knockout mice compared to WT [57], and using samples from human patients with HCM mutations in MyBP-C, a positive correlation was found between the expression of cMyBP-C and the proportion of myosin heads in the SRX state [56]. Demembranated myocardial preparations from WT and cMyBP-C null mice were studied by low-angle X-ray diffraction, and ablation of cMyBP-C appeared to result in radial displacement of myosin heads away from the thick filaments in relaxed myocardium, consistent with heads being released from a sequestered state [19]. Examination of the effects of adding C1-C2 to demembranated trabeculae from rat ventricles using fluorescent probes on the RLC also suggested that the N-terminal region of MyBP-C stabilizes the off state of thick filaments [43]. All these results are consistent with the hypothesis that cMyBP-C normally acts to tether myosin cross-bridges nearer to the thick filament backbone, holding them in a sequestered state. Haploinsufficiency of MyBP-C would therefore be expected to result in a shift of heads from the off state to the on state, explaining the hypercontractility seen clinically for MyBP-C HCM mutations.

The concept that there is an SRX-sequestered state that is highly regulated in cardiac muscle also holds true for skeletal muscle (for review, see [39]). The behaviors of the SRX population in cardiac fibers, however, are distinctly different from those in skeletal muscle fibers [38] (see “Discussion”).

### Small molecules binding to the catalytic domain favor different conformational states of myosin and alter the number of myosin molecules functionally accessible for interaction with actin

**Fig. 4:**
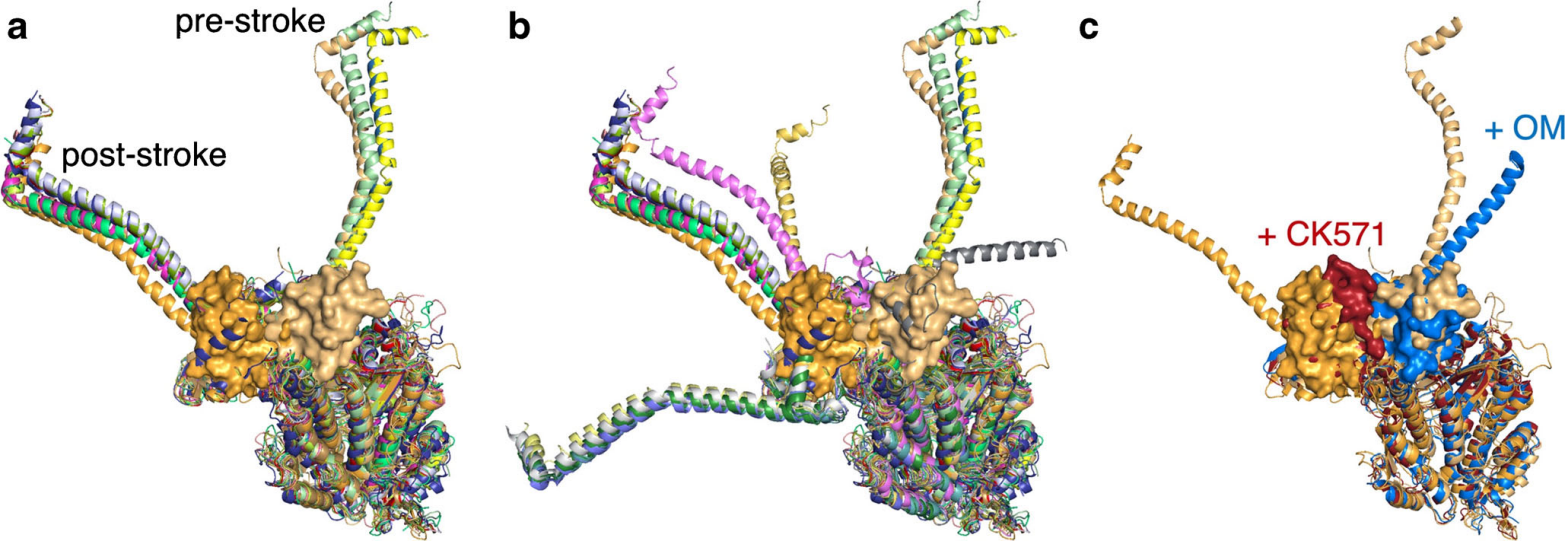
Differing conformations of S1 depending on what ligands are bound. **a** Nine X-ray crystal structures of the post-stroke state (PDB files 5H53 (skeletal muscle, dark blue), 3I5G (squid, light blue), 315I (squid, red), 3I5I (squid, smudge green), 1SR6 (scallop, lemon green), 1DFK (scallop, pink), 2EC6 (scallop, blue-green), 1S5G (scallop, gold), and 1KK7 (scallop, salmon)), and 3 X-ray crystal structures of the pre-stroke state (PDB files 1DFL (scallop muscle, marine blue), 1EFL (scallop, yellow), and 1QVI (scallop, light green). The HBCprestrokeS1 (cardiac homology model as in Fig. 3; light orange with converter depicted in PyMol surface mode) and the HBCpoststrokeS1 (cardiac homology model, orange with converter depicted in PyMol surface mode) homology models are also shown. All structures and models were aligned using residues from the beginning of the heavy chain N-termini and ending just before the converter domain. **b** The structures in **a** plus PDB files 1B7T (scallop, light yellow), 3I5F (squid, bright pink), 2MYS (skeletal, yellow gold), 1KK8 (scallop, light gray), 1KQM (scallop, medium blue), 1KWO (scallop, forest green), 1L2O (scallop, dark teal), and 1BR1 (scallop, medium gray), aligned as in (**a**). **c** The HBCprestrokeS1 (cardiac homology model, light orange) and the HBCpoststrokeS1 (cardiac homology model, orange) plus PDB files 5T45 (smooth muscle with CK571 bound, dark red) and 5N69 (cardiac with Omecamtiv mecarbil (OM) bound, medium blue)

That sS1 can exist in an SRX state is made particularly obvious when small molecules are bound to sS1 and the majority of the heads in the population show the SRX level of ATPase. Different small molecules have different effects on the functional parameters of myosin and trap the molecule in different conformational states. Figure 4a shows 12 different myosin crystal structures aligned by their catalytic domains. Three are with ATP or ADP. Pi analogs bound and have their lever arms in what is conventionally considered to be the pre-stroke state. Nine are with ADP bound or with no nucleotide in the active site and have their lever arms in what is conventionally considered to be the post-stroke state. Our complete human β-cardiac myosin homology models of the pre-stroke and post-stroke states are based on these two states of the molecule (Fig. 4a, light orange with its converter shown in surface mode as pre-stroke, and dark orange with its converter shown in surface mode as post-stroke; downloadable at http://spudlab.stanford.edu/homology-models/). There are many crystal structures of myosin, however, with their lever arms in positions that are different from the conventional pre-stroke and post-stroke configurations (Fig. 4b), showing considerable flexibility of the lever arm position. At least one intermediate position between the conventional pre-stroke and post-stroke configurations was also defined from dynamic studies on myosin using time resolved fluorescence energy transfer [76].

Just as different nucleotide states establish different conformations of the S1 head with lever arms in different positions, other small molecules, binding to sites other than the catalytic site, also position the S1 head into particular conformations. Thus, CK571, a small-molecule inhibitor of smooth muscle myosin developed by Cytokinetics, binds to the catalytic domain of myosin in an allosteric pocket near the converter domain between the relay helix and the SH1 helix, two structural elements that control the lever arm position [77]. The binding site of CK571 only becomes accessible to the small molecule in the transition between the post-stroke and pre-stroke states of the molecule, and the S1 head appears to become trapped in between the post-stroke and pre-stroke states, in this case nearer the post-stroke lever arm position (Fig. 4c, compare the position of the post-stroke converter shown in dark orange and the converter of the CK571-bound catalytic domain shown in dark red). This CK571-bound S1 has a reduced ATP turnover rate, that of the SRX. The molecule interferes with the normal relay helix-SH1 helix interactions involved in the post-stroke to pre-stroke transition [77]. This agent blocks the myosin from proceeding through its ATPase cycle and thereby eliminates its force-producing capability.

**Fig. 5:**
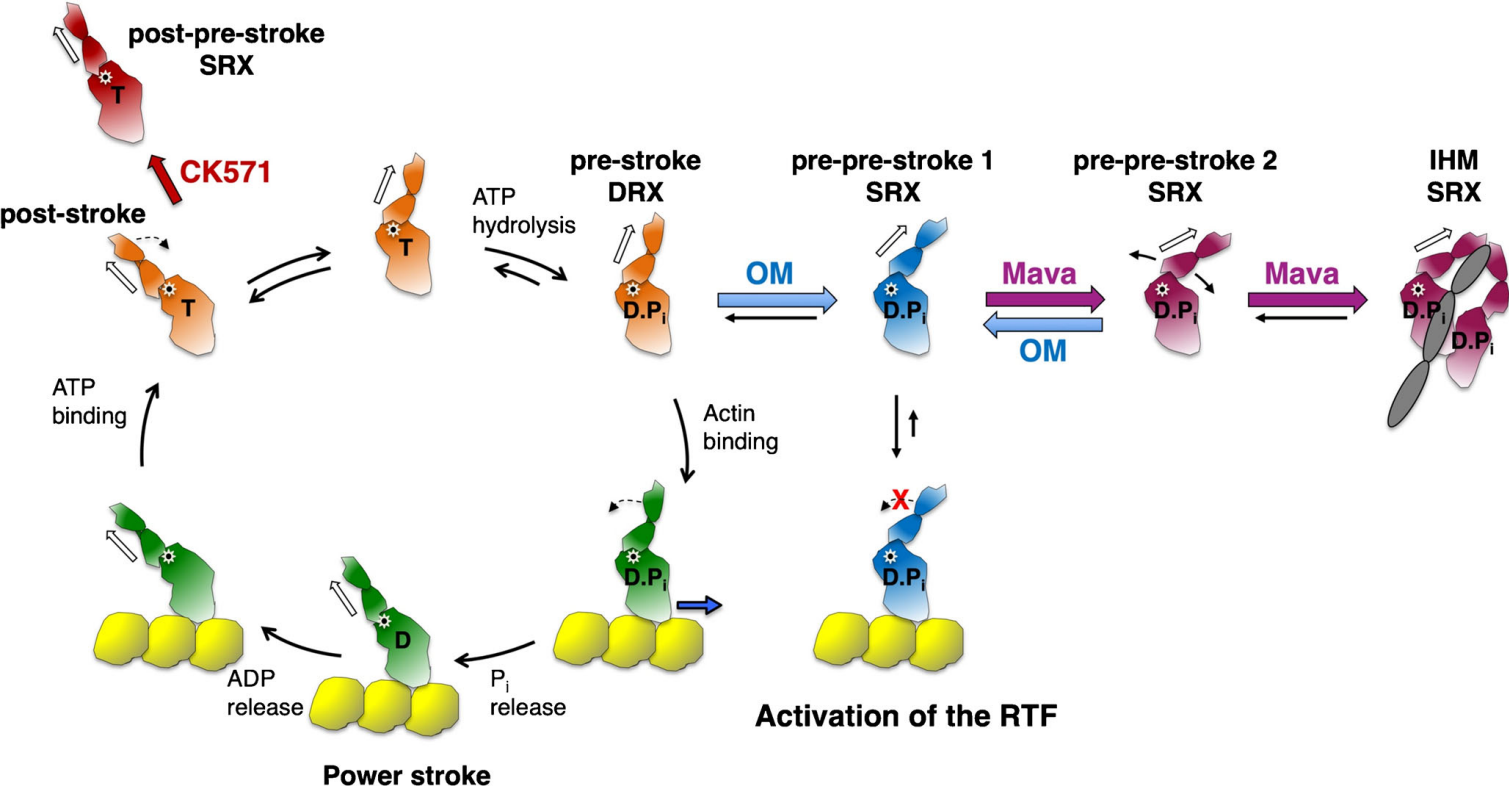
Chemo-mechanical cycle of the interaction of myosin heads with actin and off-cycle structures with small-molecule ligands bound. CK571-bound S1 (red) in a near post-stroke state. OM-bound S1 (medium blue) in a near pre-stroke state. Mavacamten (Mava)-bound S1 (purple) in a hypothetical pre-pre-stroke state

Omecamtiv mecarbil (OM), also developed by Cytokinetics and now in phase 3 clinical trials for heart failure, is an activator of cardiac muscle contraction [17, 53, 85, 86]. Part of the molecule binds near the SH1 helix, and the remainder binds close to the surface of the S1 near the converter domain [64]. OM accelerates P_i_ release in single-turnover experiments [53], and the crystal structure of OM-bound myosin heads shows them in a primed position close to that of the pre-stroke state [64]. One view of activation of myosin by OM is that the drug results in accumulation of cardiac myosin in the primed pre-stroke state prior to onset of cardiac contraction, and the resulting increase in the number of heads that can bind to the actin filament and undergo a powerstroke once the cardiac cycle starts results in higher power output [64]. However, OM slows the rate of the powerstroke [70, 101] and increases dramatically the strongly bound state time to actin [51, 101], which manifests itself as a potent inhibitor of velocity of actin filaments in vitro [4, 51, 52, 84]. OM also causes decreased isometric force in fully activated rat cardiomyocytes, using OM concentrations much higher than normal clinical doses [44]. Measurement of the basal rate of ATPase activity of OM-bound myosin in the absence of actin is that of the SRX [53]. Another view of activation of cardiac contractility by OM is that it pulls the equilibrium away from the folded-back sequestered off state and stabilizes the head in a pre-pre-stroke state (defined as a state with its lever arm bent further in the pre-stroke direction than the conventional pre-stroke conformation; see Fig. 4c and Fig. 5), a state not compliant enough to fit into the folded-back sequestered state [68] and still able to bind to actin (Fig. 5). According to this view, OM activates the sarcomere at clinically relevant doses by a small number of OM-bound S1 heads enhancing the movement of tropomyosin-troponin (Tm.Tn) to the on state of the regulated thin filament (actin.Tm.Tn) [51, 101], as well as possibly reducing the number of heads in the folded-back sequestered off state (Fig. 5).

The effects of OM are in sharp contrast to those of the small-molecule myosin inhibitor blebbistatin. Blebbistatin is an inhibitor of myosin II which maintains the helical order of myosin heads on thick filaments (the off state) [103]. This result is in keeping with the observations that blebbistatin induces the SRX low-ATPase state of myosin and causes spin-labeled nucleotides bound to myosin to have an oriented spectrum in skeletal muscle fibers, consistent with highly ordered heads in their off state [98]. Kampourakis et al. [44] came to the same conclusion in studies with demembranated cardiac muscle cells. They measured isometric force in fibers, ATP utilization in homogenized myocardium, and in situ structural changes using fluorescent probes on the myosin RLC in the thick filaments, and they showed that blebbistatin reduces contractility by stabilizing the thick filament off state and inhibiting actin-activated myosin ATPase.

Mavacamten (Mava), an inhibitor of cardiac contraction being developed by MyoKardia for treatment of HCM, also in phase 3 clinical trials, binds to sS1 and induces an SRX level of basal ATPase [9, 46, 69], just like CK571 and OM. Unlike OM, however, Mava appears to position the S1 in a structural state that easily fits into a folded-back sequestered state [9], possibly the IHM configuration. Either the Mava-bound S1 takes up a head configuration that is very near the head configurations in the folded-back sequestered state (Fig. 5), or it has the compliance to easily adopt the conformation(s) of the heads in the folded-back sequestered state. As Houdusse and her colleagues have shown [68], such compliance may be modified, for example, by the positions of loops in the converter domain that affect the dynamics of the converter/ELC interface. The result of Mava binding to bovine cardiac HMM [69] and human β-cardiac 25-hep HMM [9] is inhibition of the basal ATPase to the SRX level and a total inhibition of actin-activated ATPase activity [46].

The difference between OM and Mava may be that the OM-bound S1 conformation cannot enter the folded-back sequestered state and therefore acts as an activator of contractility, while the Mava-bound S1 conformation has the proclivity to enter the folded-back sequestered state and is therefore removed from the functionally accessible pool of molecules for actin interaction. This may mean that one can convert an activator to an inhibitor with very small changes in the structure of a small molecule, and vice versa.

In favor of perspective 2 is that it offers a simple and potentially unifying view as to how most HCM mutations, both in myosin and MyBP-C, cause the hypercontractility seen clinically, and experimental data are accumulating that so far have not refuted this concept (as scientists, we strive to disprove our hypotheses), but rather have lent support to it.

## The third perspective: load dependence of contractility is affected by cardiomyopathy mutations and small-molecule effectors in a manner that changes the power output of cardiac contraction

**Fig. 6:**
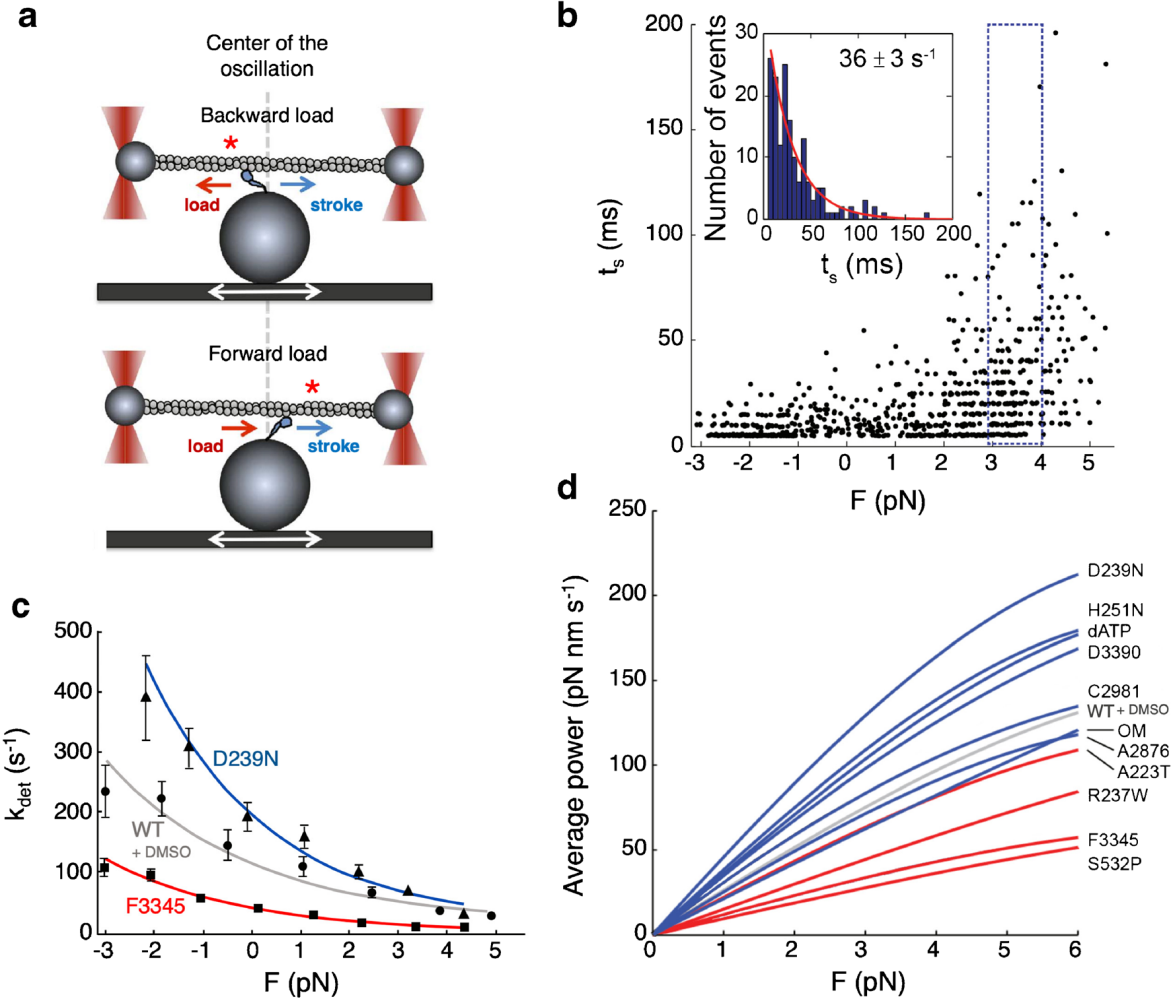
Single-molecule load dependence measurements using harmonic force spectroscopy. **a** Schematic illustration of the harmonic force spectroscopy technique. The myosin can bind to the actin filament at any position of the oscillating stage, which produces a range of backward and forward loads exerted on the myosin molecule. **b** Plot of the strongly bound state time (*t*_s_) of WT human β-cardiac sS1 bound to actin as a function of backward (resistive, positive force (*F*) values) and forward (assistive, negative *F* values) load. The insert shows the number of events as a function of *t*_s_ derived from the data from the 1-pN bin shown by the dashed rectangle (from + 3 to + 4 pN). Fitting the data to a single exponential gave a detachment rate constant (*k*_det_) of 36 ms. **c** Plot of *k*_det_ as a function of the load (*F*) for the HCM mutant D239N human β-cardiac sS1 (blue), WT human β-cardiac sS1 with the small-molecule inhibitor F3345 bound (red), and WT human β-cardiac sS1 in 2% DMSO (small-molecule experiments were carried out in 2% DMSO; the WT without DMSO curve was very similar). **d** Plot of the average power output as a function of load (*F*) for WT human β-cardiac sS1 with small-molecule activators bound and 2 HCM sS1 mutants (blue), and human β-cardiac sS1 with a small-molecule inhibitor bound, and 3 DCM sS1 mutants (red). The WT + DMSO control is shown in light gray

Neither of the first two perspectives takes into account the well-known load dependence of muscle contractility [25, 26, 65]. Superimposed on any effects of mutations or small molecules on the parameters discussed in the previous sections are possible effects on the load dependence of myosin function. To measure load dependence in vitro with purified proteins, one can use harmonic force spectroscopy [83], which makes use of a dual-beam laser trap with an oscillating stage. If myosin binds to actin when the stage is to the left of center in Fig. 6a top (e.g., near the red star), then as the stage continues to oscillate, the myosin experiences a net backward, resistive load, whereas if myosin binds when the stage is to the right of center, then as the stage continues to oscillate ,the myosin experiences a net forward, assistive load (Fig. 6a bottom). Since the myosin can bind anywhere along the actin during the oscillation, one obtains data points for the strongly bound state time (*t*_s_) as a function of load between − 3 and + 5 pN, as shown in Fig. 6b.

The data for each pN segment is then binned, as shown in the 3 to 4 pN example for WT human β-cardiac sS1 in Fig. 6b, and the number of events is plotted as a function of the *t*_s_ (see insert Fig. 6b). From this, a single exponential rate is determined and plotted as the detachment rate (*k*_det_) as a function of force (*F*) (Fig. 6c). When the data from all of the bins in Fig. 6b are plotted for the WT human β-cardiac sS1 molecule, one obtains the load dependence of the detachment rate (Fig. 6c). Such curves are obtained for many individual WT molecules and they are averaged to give a single WT curve (Fig. 6c, gray). Figure 6c also shows data from D239N HCM-sS1 (blue) and F3345-bound human β-cardiac sS1 (red) to illustrate that mutations and small molecules can indeed change the load dependence of *k*_det_ quite significantly. When these data for a number of cardiomyopathy mutations and small-molecule effectors, including OM, were combined with actin-activated ATPase data, the average power output could be calculated as a function of load [51]. The result was a grouping of all activators/HCM mutations showing higher power output as a function of force (blue curves) than the grouping of all inhibitors/DCM mutations (red curves) (Fig. 6d).

In a complementary study, Woody et al. [101] used a feedback-controlled laser trap to show that OM suppresses the myosin working stroke and prolongs the actin-attachment lifetime at physiological ATP concentrations. Both the Woody et al. [101] and Liu et al. [51] studies are consistent with a model in which myosin is not able to produce a working stroke with OM bound but is still able to interact with actin, probably activating the regulated thin filament. Furthermore, the mechanistic effects of OM are the same at all OM concentrations, and the effects are simply a summation of what is expected from varying ratios of OM-bound heads and OM-free heads [51].

## Conclusions

The data from all three perspectives inform on the molecular basis of hypercontractility caused by HCM mutations. Some HCM mutations, such as D239N and I457T, cause increases in ATPase, velocity, and *F*_int_ that easily account for the hypercontractility seen clinically. The position of these mutations in the IHM model of the folded-back sequestered state is not in predicted intramolecular interaction sites. Many other HCM mutations that have been studied, however, are in predicted intramolecular interaction sites of the IHM and data to date support those mutations weakening the sequestered state and increasing the number of accessible heads for interaction with actin, giving rise to the hypercontractility seen clinically. Indeed, increasing *N*_a_ may be a unifying hypothesis for hypercontractility due to most HCM mutations in both myosin and MyBP-C [2, 7, 60, 68, 79, 80, 88]. Load dependence is also important and the load-dependent power output compared to WT increased significantly for the two HCM mutations studied (H251N and D239N) [51]. Kinetic studies to estimate changes in the population of the A·M·D state showed an increase for R453C and no change for R403Q, although the authors suggested that if one takes into account the expected effects of load, R403Q myosin would also show an increase in the population of the A·M·D state [90]. Thus, one must consider all three perspectives to understand the hypercontractility seen clinically, with the mesa hypothesis possibly playing a dominant role in the ~ 70% of HCM mutations in the two sarcomeric proteins β-cardiac myosin and MyBP-C.

Some of you will say upon reading this review that the usual clinical description of hypertrophic cardiomyopathy is impaired diastolic function with preserved systolic function, rather than overt hypercontractility. It is possible that hypercontractility will be more the norm as more genotype-positive HCM patients are examined compared to controls in early-stage patients. Such early measurements have not received a lot of attention in the field. But also consider the following. My working hypothesis is that all three perspectives described here lead to HCM-mutation-induced increases in the number of myosin heads interacting with actin, or an increase in the strongly bound state time, or an increase in load-dependent power output. All of these changes will potentially impact on the Tm.Tn system, activating the thin filament. This thin filament activation will contribute to hypercontractility of the heart. As described in this review, I believe that most myosin and MyBP-C HCM mutations will be shown to result in an increase in the number of functionally accessible myosin heads (*N*_a_) for interaction with actin (perspective 2). This increase in *N*_a_ would account for the observed diastolic dysfunction due to the increase in the number of myosin heads that must be released from the actin as the muscle goes into diastole. Interestingly, to our great surprise, our observations of the effects of myosin HCM mutations on the fundamental parameters of ATPase activity, velocity, and intrinsic force (perspective 1) have often appeared to result in a net hypocontractility (e.g., see the R403Q studies of Nag et al. [59]), or borderline hypocontractility/hypercontractility. It is possible that HCM-mutation-induced hypocontractile contributions from decreases in ATPase activity, velocity, and/or intrinsic force may often offset the hypercontractility derived from the increase in *N*_a_, resulting in preservation of systolic function rather than overt hypercontractility. Nonetheless, the increase in *N*_a_ will still manifest itself as prolonging diastole. Thus, it remains an open question what is triggering the clinical disease phenotypes of hypertrophy, fibrosis, and myofilament disarray. Is the heart responding to an HCM-mutation-induced overall increase in power output, or is the signal coming from the Tm.Tn Ca^2+^ regulatory system, which arguably is where the molecular effects of all HCM mutations may be funneled, or is there some other primary event that acts as the fundamental early signal for induction of the characteristic clinical manifestations of HCM? These are some of the most fundamental unanswered questions in the field.

Many other issues remain unresolved. A pressing need is to obtain the high-resolution structure of the folded-back sequestered state of human β-cardiac HMM and at least a low-resolution structure of the MyBP-C-bound human β-cardiac HMM. Another important issue is that most studies have involved the C0-C2 region of MyBP-C, and it is imperative to explore whether and how the C3-C7 domain may affect MyBP-C interaction with myosin. An additional pivotal question is what regulates MyBP-C interaction with myosin versus actin, since binding to both appears to be physiologically significant and complementary, where myosin binding is inhibitory for contraction and actin binding is activating.

Another issue in need of study is the kinetics of the equilibrium between the sequestered off state of myosin and its open on state. One extreme would be that in the heart, the transition between the two states occurs in milliseconds, with all the myosin heads being released during systole and all the heads being parked against the thick filament in the off state during diastole, the switch between them being the presence of systolic levels of Ca^2+^. The other extreme would be that many myosin heads in the heart are parked in the off state for longer time periods and are heads held in reserve to be activated by phosphorylation or other signals to release them into the active pool when more power output is needed. Phosphorylation of the RLC by MLCK, for example, is known to favor the open state in both cardiac and skeletal muscles [21, 42, 60, 63, 87]. The SRX measurements in skinned cardiac fibers by Cooke and his colleagues suggest that many myosin heads in the heart are parked in the off state for longer time periods and are heads held in reserve [38]. They found that ~ 50% of the myosin heads are in the SRX state in cardiac muscle fibers and this value did not change upon activation of the fibers with Ca^2+^. Purified dephosphorylated human β-cardiac HMM shows a distribution of ~ 40% SRX and ~ 60% DRX in 25 mM potassium acetate and these are clearly not in rapid equilibrium since biphasic kinetics is seen over a period of several hundred seconds [9].

Skeletal muscle fibers may be a different story however. Hoojiman et al. [38] showed that the SRX in relaxed skeletal muscle fibers completely disappeared upon activation of the fibers with Ca^2+^. Thus, the kinetics of the equilibrium between the SRX and DRX states may depend on the muscle type. This also suggests that the effects of Ca^2+^ on the distribution of SRX and DRX states need to be fully explored for both skeletal and cardiac muscle myosins, both in fibers and as purified proteins.

The story becomes more complicated but interesting when considering that ~ 15% of the myosin heads in the heart are likely sequestered by MyBP-C. As already mentioned, it is not known whether MyBP-C is further stabilizing the normal sequestered state, probably the IHM, or is sequestering heads in a different state. Further sequestration of the normal sequestered state would seem to park MyBP-C-bound myosin heads into a more stable state, with heads in reserve that would only be released by both phosphorylation of MyBP-C [41] and of the RLC. If one considers a different sequestered state, however, then other functional options need to be considered. For example, the MyBP-C-sequestered heads could be more accessible for entering the active myosin head pool, with the MyBP-C facilitating the head interaction with actin while simultaneously activating the thin filament [91].

Other issues that need to be addressed include the roles of titin, if any, in regulating the number of heads in the SRX state, and what interactions occur between the HMM portion of myosin and the LMM core of the thick filament that keeps myosin heads sequestered tightly against the thick filament backbone. Furthermore, is there more than one SRX state? The definition of the SRX state by Cooke and his colleagues is a state with a very low turnover rate of ATP of ~ 0.003 s^−1^ [38]. Anderson et al. [9] showed with purified human β-cardiac HMM that a folded-back sequestered state, probably the IHM, is in the SRX state according to this definition. It has also been shown, however, that the myosin head alone can also be in this SRX state [9, 69]. Therefore, there is not just a single structural state corresponding to the SRX. Furthermore, one should consider that there may be a series of different structural states of the myosin with differing ATP turnover rates, including a possible augmented-super-relaxed state with an even lower ATPase turnover rate (< 0.001 s^−1^). Thus, there is much to be done to understand the fascinating regulation of thick filament behavior and function.
